# Murine cartilage microbial DNA deposition occurs rapidly following the introduction of a gut microbiome and changes with obesity, aging, and knee osteoarthritis

**DOI:** 10.1007/s11357-023-01004-z

**Published:** 2023-11-09

**Authors:** Vladislav Izda, Leoni Schlupp, Emmaline Prinz, Gabby Dyson, Montana Barrett, Christopher M. Dunn, Emily Nguyen, Cassandra Sturdy, Matlock A. Jeffries

**Affiliations:** 1 Oklahoma Medical Research Foundation, Arthritis & Clinical Immunology Program, 825 NE 13th Street, Laboratory MC400, Oklahoma City, OK 73104 USA; 2 Icahn School of Medicine, Mt. Sinai, New York, NY USA; 3 Department of Internal Medicine, Division of Rheumatology, Immunology, and Allergy, University of Oklahoma Health Sciences Center, Oklahoma City, OK USA; 4 VA Medical Center, Oklahoma City, OK USA

**Keywords:** Osteoarthritis, Mouse models, Microbiome, High-fat diet, Aging

## Abstract

Cartilage microbial DNA patterns have been recently characterized in osteoarthritis (OA). The objectives of this study were to evaluate the gut origins of cartilage microbial DNA, to characterize cartilage microbial changes with age, obesity, and OA in mice, and correlate these to gut microbiome changes. We used 16S rRNA sequencing performed longitudinally on articular knee cartilage from germ-free (GF) mice following oral microbiome inoculation and cartilage and cecal samples from young and old wild-type mice with/without high-fat diet-induced obesity (HFD) and with/without OA induced by destabilization of the medial meniscus (DMM) to evaluate gut and cartilage microbiota. Microbial diversity was assessed, groups compared, and functional metagenomic profiles reconstructed. Findings were confirmed in an independent cohort by clade-specific qPCR. We found that cartilage microbial patterns developed at 48 h and later timepoints following oral microbiome inoculation of GF mice. Alpha diversity was increased in SPF mouse cartilage samples with age (*P* = 0.013), HFD (*P* = 5.6E-4), and OA (*P* = 0.029) but decreased in cecal samples with age (*P* = 0.014) and HFD (*P* = 1.5E-9). Numerous clades were altered with aging, HFD, and OA, including increases in *Verrucomicrobia* in both cartilage and cecal samples. Functional analysis suggested changes in dihydroorotase, glutamate-5-semialdehyde dehydrogenase, glutamate-5-kinase, and phosphoribosylamine-glycine ligase, in both cecum and cartilage, with aging, HFD, and OA. In conclusion, cartilage microbial DNA patterns develop rapidly after the introduction of a gut microbiome and change in concert with the gut microbiome during aging, HFD, and OA in mice. DMM-induced OA causes shifts in both cartilage and cecal microbiome patterns independent of other factors.

**Supplementary Information:**

The online version contains supplementary material available at 10.1007/s11357-023-01004-z.

## Introduction

Osteoarthritis (OA) is a chronic, age-associated musculoskeletal disease characterized by progressive loss of function of joints leading to pain, mobility loss, significant morbidity, and early mortality. It is the leading cause of chronic disability in the USA, affecting roughly half of adults over 65 years of age [1]. Despite its impact, there are no disease-modifying drug therapies available, due in no small part to an incomplete understanding of OA pathogenesis.

One potential OA pathogenic factor is the microbiome. The gut microbiome in both humans and mice changes with aging and obesity, two key non-genetic risk factors for OA [2, 3]. Although expanding, the field of OA microbiomics research is still limited. The largest human study to date identified four bacterial clades associated with knee pain among 867 adults in the Netherlands, including class *Bacilli*, order *Lactobacillales*, family *Streptococcaceae*, and genus *Streptococcus* [4]. In mice, induction of obesity by a high-fat diet leads to shifts in the microbiome including reductions in the *Bifidobacterium* species and increases in abundance of *Peptostreptococcaceae* species, both associated with obesity and intestinal inflammation [5–7], and both associated with acceleration in OA severity after destabilization of the medial meniscus (DMM) surgery. Supplementation of the mouse diet with the oligofructose reverses obesity-related gut microbial changes, increasing *Bifidobacteria* and reducing *Peptostreptococcaceae* within the gut and associated reductions in circulating lipopolysaccharide levels and OA pathological changes following DMM.

To further elucidate the microbial shifts associated with OA, our laboratory recently published the first detailed description of microbial DNA within human cartilage [8]. We found substantial shifts in cartilage microbial DNA patterns when comparing diseased human OA tissues to disease-free controls, including a loss of alpha diversity, enrichment in Gram-negative constituents, and shifts in a variety of microbial clades. Other studies have similarly identified bacterial DNA in synovial fluid [9] and synovial tissue [10]. However, the source of these cartilage microbial traces and whether these patterns are fixed or change along with the gut microbiome following perturbations of various environmental factors is unknown.

In the present study, we hypothesized that cartilage microbial DNA is sourced from gut microbiota, aging and obesity would be associated with shifts in both cartilage and cecal microbial DNA patterns, and these changes would mirror OA-associated microbiome shifts in both niches. To evaluate this, we first performed a longitudinal analysis of cartilage microbial DNA development in germ-free (GF) mice following oral microbiome inoculation. Next, we performed cartilage and cecal microbiome composition analysis via 16S rRNA next-generation sequencing of mice under various aging, dietary, and OA conditions.

## Materials and methods

### Ethics statement, experimental unit

The institutional animal care and use committee of the Oklahoma Medical Research Foundation (OMRF) approved this study (OMRF IACUC protocol numbers 16-40, 19-43, 20-29, 19-56, 18-45, 18-18). The experimental unit was a single animal.

### Animal diets

The chow diet used was the PicoLab Rodent Diet 20 (LabDiet #5053) and consists of 4.7% crude fiber, 5.0% fat (ether extract), 5.6% fat (acid hydrolysis), and 20.0% protein; caloric content was 25.7% from protein, 13.2% from fat, and 62.1% from carbohydrate. The high-fat diet (HFD) used was the Research Diets D12492 diet, with 60% of caloric content from fat, consisting of 6.5% crude fiber, 35% fat (32% from lard, 3.2% from soybean oil), and 26% protein.

### Germ-free mouse inoculation experiments

Germ-free (GF) C57BL6/N mice were bred and maintained in the Rodent Gnotobiotic Core facility at the Oklahoma Medical Research Foundation. At 12 weeks of age, female GF mice (only female GF mice were available at the time of our experiment) were removed from isolators and immediately inoculated via oral gavage with 200 μL of a pooled cecal transplant slurry consisting of a 1:5 dilution of freshly obtained cecal contents from 12 week-old wild-type C57BL6/J male specific pathogen free (SPF) mice, diluted in a 1:1 mixture of sterile PBS and glycerol. Mice were immediately transferred into sealed positive-pressure cages (Sentry SPP) to prevent contamination, fed the same irradiated chow as GF animals, and sacrificed at predetermined timepoints after transplantation including 4 h (*n* = 6), 24 h (*n* = 6), 48 h (*n* = 6), 1 week (*n* = 6), 2 weeks (*n* = 6), and 4 weeks (*n* = 6). Uninoculated GF mice were used as controls (*n* = 10). Cartilage was processed as below and 16S sequencing reads per knee sample for each mouse calculated.

### Specific pathogen free (non-germ-free) mouse experiments

Young (12 weeks of age) and old (18 months of age) C57BL6/J male mice were fed either chow or HFD (60% kcal from fat) for 8 weeks prior to euthanasia. In a subset of young chow animals, DMM surgery was performed on a unilateral stifle (knee) joint at 16 weeks of age then sacrificed 4 weeks later. Female mice were excluded, as only male mice reliably exhibit an OA phenotype following destabilization of the medial meniscus (DMM) surgery [11]. All animals were permitted access to food and water ad libitum and were exposed to a 12-h light-dark cycle. All animal husbandry procedures adhered to the NIH Guide for the Care and Use of Laboratory Animals. There were no unexpected adverse events during these experiments. HFD and DMM animals were randomly assigned from litters. Animals segregated by age and diet group, up to 5 mice were cohoused in the same cage. Animals fed a HFD weighed significantly more at sacrifice than chow animals (young chow, *n* = 6, 27.6 ± 0.5g mean ± SEM, young HFD, *n* = 6, 37.3 ± 1.3g, *P* < 0.0001), (old chow, *n* = 6, 42.7 ± 2.5 g, old HFD, *n* = 6, 62.4 ± 2.8 g, *P* = 0.0003), Supplementary Figure 1. To ensure aged mice did not develop incidental OA that could bias our results, additional age-matched cohorts of young B6 (*n* = 5), old B6 (*n* = 5), and young B6 + DMM (*n* = 5) mice were generated for histologic, osteophyte, and synovial hyperplasia/synovitis scoring using the OARSI recommendations. No difference was seen between young and old non-DMM mice from an OARSI histopathologic (young 0.6 ± 0.2 vs. old 0.6 ± 0.1, mean ± SEM, *P* = 0.9), osteophyte (young 0.1 ± 0.06 vs. 0.1 ± 0.1, *P* = 0.8), nor synovitis (young 0.58 ± 0.1 vs. 0.58 ± 0.08, *P* = 1.0) scoring perspective, Supplementary Figure 2. One old HFD cartilage sample, 2 old chow cecal samples, 1 young HFD cecal, and 1 young chow + DMM cecal samples were excluded due to failed amplification and/or 16S sequencing.

### Sample processing

Knee joints were dissected in a biosafety cabinet using sterilized, UV- and DNA/RNA-decontaminated (DNA-Zap solution, ThermoFisher, Waltham, MA, USA) instruments following skin and synovial capsule sterilization with chlorhexidine. Full-thickness articular cartilage was removed from the tibia and femur using a disposable, sterile #11 blade and immediately flash frozen and stored in liquid nitrogen. Later, cartilage samples were cryogenically ground using a Precellys Cryolys instrument (Bertin, Bretonneux, France) at 0 °C and DNA isolated using a DNEasy kit (Qiagen). Cecal contents were flash frozen in liquid nitrogen then DNA extracted using a Qiagen QIAamp DNA microbiome kit. All plasticware and reagents were decontaminated by a 30-min UV exposure as previously described [8, 12, 13]. PCR master mixes and tubes were further enzymatically decontaminated with dsDNAse (PCR decontamination kit, Arcticzymes, Tromsø, Norway).

### Control experiments

We performed an additional control experiment to ensure the fidelity of our decontamination procedures. In this experiment, we spiked the surface of four germ-free B6 mouse hindlimbs and performed the same 16S microbial DNA analysis of cartilage as detailed below. We found no differences in diversity nor any microbiome clade differences comparing germ-free to skin-spiked germ-free animals (microbial counts were expectedly very low and consistent with background).

Then, we performed a final control experiment to ensure that our microbial findings in DMM-induced OA mice were indeed related to the development of OA rather than an effect of a surgical procedure. In this experiment, we performed sham surgery (opening skin, subcutaneous tissues, and joint capsule but not transecting the medial meniscus) on four mice, then extracted cartilage tissue and DNA 4 weeks later, as detailed above. We found no differences in diversity nor any microbiome clade differences comparing sham knees to non-operated control mouse knees (different animals), nor did we find differences when comparing sham knees to contralateral unoperated knees (same animal).

### Serum LPS analysis

A Pierce chromogenic endotoxin quantification kit was used to quantify LPS (Thermo Fisher, Waltham, MA, USA) using an amebocyte lysate method and has a sensitivity of 0.01 EU/mL and an assay range of 0.01–0.1 EU/mL. LPS-free plasticware was utilized. Endotoxin-free water was used to dilute standards and samples were diluted 1:10. All analyses were performed using 2 technical replicates. The coefficient of determination (*R*^2^) of the standard curve was 0.95. Statistical significance was defined as *P* ≤ 0.05. Inadequate serum was available for evaluation in 7 mice: 1 young B6-chow, 2 young B6-HFD, 1 old B6-chow, 2 old B6-HFD, 1 young B6-DMM.

### 16S ribosomal RNA (rRNA) gene sequencing

Microbial profiles were determined by sequencing a ~460 bp region including the V3 and V4 variable regions of bacterial 16s rRNA genes (primers in Supplementary Table 1) using a high-fidelity polymerase (NEG Q5, New England Biolabs). For longitudinal GF experiments, 2 μL of DNA per joint was used as PCR input. For cecal experiments approximately 30 ng of DNA was used as input from each sample. Illumina Nextera XT indices were attached, pooled in equimolar amounts, and sequenced on an Illumina miSeq sequencer using a 250 bp paired-end sequencing protocol by the Clinical Genomics Center at OMRF. Four cecal samples (2 old chow, 1 old HFD, 1 young chow + DMM) and 1 cartilage sample (old HFD) were excluded from analysis due to failed PCR amplification and/or 16S sequencing. No GF cartilage samples were excluded from analysis.

### 16S rRNA OTU classification

Quality filtering, operational taxonomic unit (OTU) classification, and microbial diversity analysis were performed using the Quantitative Insights into Microbial Ecology (QIIME) software package, version 1.9.1 [14]. Sequences were assigned to OTUs using the UCLUST algorithm [15] using a 97% pairwise identity threshold and taxonomy assigned using the GreenGenes 13_8 database [16].

### Diversity analyses

Alpha diversity was characterized using the observed OTUs method following rarefaction to the lowest number of OTUs present per group. Beta diversity was evaluated on a variance-adjusted, weighted unifrac model. An adonis (permuted analysis of variance, a multi-factor PERMANOVA) test with 999 permutations was used to calculate statistical significance of difference among the 5 mouse groups [17, 18]. Unsupervised clustering was performed using a Euclidean distance matrix and the hierarchical clustering function of R.

### Group analyses

Group analyses were performed using the linear discriminant analysis effect size (LEfSe) pipeline [19]. LEfSe performs a non-parametric Kruskal-Wallis sum-rank test [20] to detect features with significant differential abundance between groups, *P* values ≤ 0.01 were considered significant. Next, it uses a linear discriminant analysis (LDA) [21] to estimate the effect size of each differentially abundant feature. An LDA threshold of ≥ 2 (corresponding to *P* ≤ 0.01) was considered significant [22]. Given the exploratory nature of the present study and the stringent null hypothesis rejection inherent to the LDA step of LEfSe, FDR correction was not applied; this is in line with the initial LEfSe publication, where multiple testing correction was not considered necessary [19]. For Gram status and *Firmicutes*:*Bacteroidetes* ratio comparisons, differences were evaluated by Student *t*-tests following outlier detection with a Grubb’s test (alpha = 0.05), *P* ≤ 0.05 was considered statistically significant.

### Prediction of metagenome content and imputed bacterial functional classification

The Phylogenetic Investigation of Communities by Reconstruction of Unobserved States (PICRUSt) software package [23] was used to impute bacterial metagenomes from our 16S deep sequencing microbial DNA data, and functional annotation was applied using the Kyoto Encyclopedia of Gene and Genomes (KEGG) catalog [24]. Statistical analysis was performed using the Statistical Analysis of Metagenomic Profiles (STAMP) package [25]. Statistical significance and effect size among the 5 groups (young chow, young chow + DMM, young HFD, old chow, and old HFD) were calculated in the STAMP v.2.1.3 software package using ANOVA with a Tukey-Kramer post-hoc test (alpha = 0.95) followed by Benjamini-Hochberg (BH) multiple test correction. Effect sizes were calculated using an Eta-squared statistic. Statistical significance was defined as BH-corrected *q* ≤ 0.05.

### Clade-specific qPCR confirmation cohort

Knee cartilage and cecal contents from an independent confirmation cohort of 6 young chow, 6 young HFD, 6 old chow, and 6 young chow + DMM animals were obtained as above. Clade-specific quantitative PCR (qPCR) analysis was performed to calculate the relative presence of *Bacteroidetes* [26], *Lactobacillales* [27]*, Turicibacteriales* [28], *Streptococcaceae* [29], *Alcaligenaceae* [30], and *Verrucomicrobia* [26] in each sample compared to a universal bacterial primer set (primers in Supplementary Table 1) using a Luna qPCR kit (New England Biolabs) on a RotorGeneQ (Qiagen) instrument. Relative clade composition was calculated using the delta-delta CT method [31]. Group differences were calculated with a Student *t*-test, *P* ≤ 0.05 was considered statistically significant.

## Results

### Cartilage microbial DNA patterns develop 48 h after introduction of a gut microbiome into GF mice

**Fig. 1:**
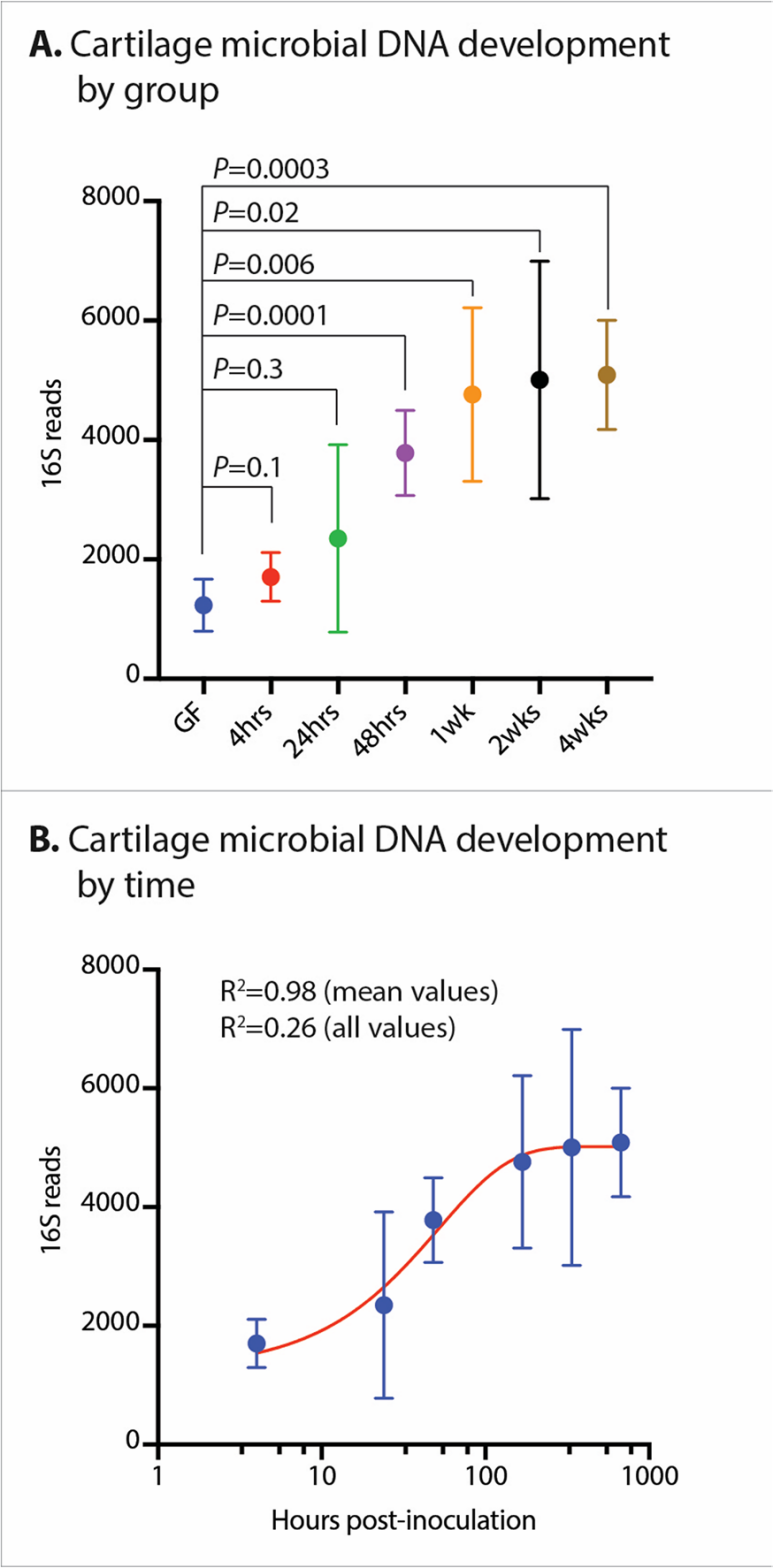
Longitudinal development of cartilage microbial DNA profiles following inoculation of an oral microbiome into germ-free (GF) mice. **A** 16S sequencing reads by mouse group. “GF” denotes uninoculated GF control animals. Time indicated is post-inoculation of GF animals with cecal microbiota from wild-type B6 mice. **B** 16S sequencing reads by hours post-inoculation of GF animals, horizontal axis logarithmic scale. Regression curve fitted using an exponential plateau model

**Fig. 2:**
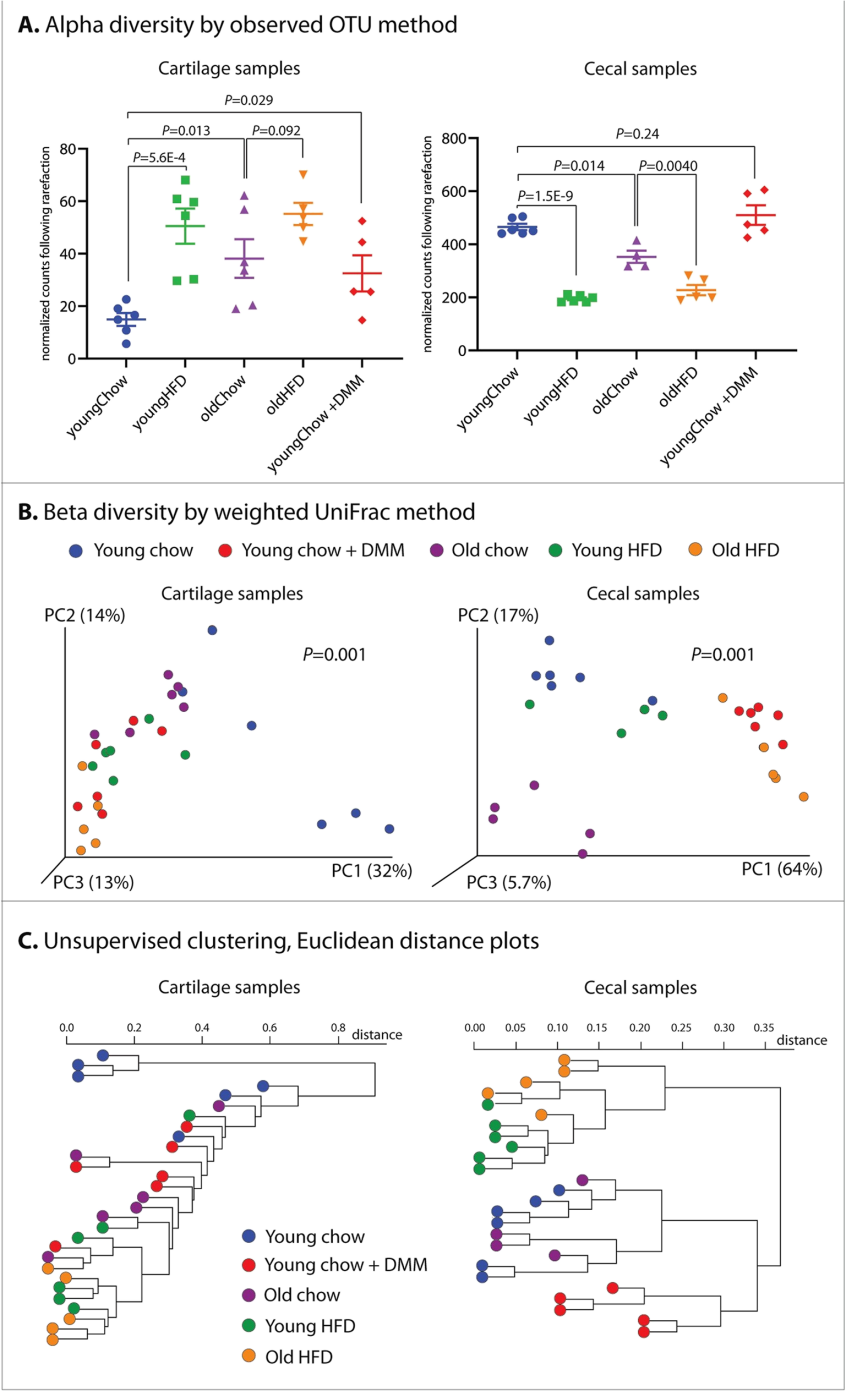
Diversity measures and clustering of cartilage and cecal microbial DNA profiles of mice under various aging, diet, and OA conditions. Young = 12 weeks of age, old = 18 months of age, HFD = 8 weeks of high-fat diet treatment, +DMM = 4 weeks after disruption of the medial meniscus surgery, inducing post-traumatic OA. **A** Alpha diversity by observed OTU method. **B** Beta diversity by weighted UniFrac method. **C** Unsupervised clustering based on 16S sequencing data using Euclidean distance plots

We first evaluated whether, and how rapidly, cartilage microbial DNA patterns develop following the introduction of a gut microbiome via oral gavage into previously GF mice. Microbial 16S reads rose to statistical significance above background at the 48-h timepoint and continued to rise through 4 weeks, when the number of 16S reads was 4.1× the background read number from control GF mice (Table 1, Fig. 1). We then determined that cartilage microbial DNA development followed an exponential plateau pattern (Fig. 2) with *R*^2^ = 0.98 (mean values per timepoint considered) and *R*^2^ = 0.26 (all values considered).

**Table 1:** Longitudinal population of cartilage microbial DNA following introduction of a gut microbiome into GF mice

Time following oral microbiome inoculation	Number (*N*)	16S reads mapped to genome (mean ± SEM)	*P* value vs. uninoculated GF control
GF negative control	10	1231 ± 1372	N/A
4 h	6	1705 ± 995	0.1
24 h	6	2348 ± 3840	0.3
48 h	6	3782 ± 1741	0.0001
1 week	6	4760 ± 3564	0.006
2 weeks	6	5005 ± 4870	0.02
4 weeks	6	5088 ± 2244	0.0003

### Serum LPS analysis suggests HFD and OA are associated with increased intestinal permeability but not aging

We next performed a serum LPS quantitation to indirectly estimate changes in intestinal permeability. We found increases in LPS with HFD in young animals (young chow 0.52 ± 0.05 vs. 0.76 ± 0.06, mean ± SEM units, *P* = 0.003) and old animals (old chow 0.59 ± 0.03 vs. 0.72 ± 0.05, *P* = 0.05), and with DMM-induced OA (young chow 0.52 ± 0.05 vs. young DMM-induced OA 0.66 ± 0.1, *P* = 0.04) but not with aging (young chow 0.52 ± 0.05 vs. old chow 0.59 ± 0.03, *P* = 0.8).

### Aging, obesity, and OA via DMM surgery are associated with increases in cartilage alpha diversity, whereas OA risk factors, though not OA itself, induce reductions in cecal alpha diversity

Our final SPF analysis included cartilage from 29 mice (6 young chow, 6 young HFD, 6 old chow, 5 old HFD, 6 young chow + DMM) and 26 cecal samples (6 young chow, 6 young HFD, 4 old chow, 5 young HFD, 5 young chow + DMM). Raw read counts are presented in Supplementary Tables 2 and 3; all samples were rarefied to the same number of raw reads before additional processing.

In cartilage samples, age, HFD, and OA were all independently associated with increases in alpha diversity compared to young non-OA samples (Fig. 2A, young chow 15.0 ± 2.5, mean ± SEM vs. old chow 38.1 ± 7.4, *P* = 0.01; young chow vs. young HFD 50.5 ± 6.7, *P* = 5.7E-4; young chow vs. young chow + DMM 32.5 ± 2.2, *P* = 0.03). There was a nonsignificant increase in alpha diversity in old HFD samples compared to old chow samples (55.2 ± 4.2 vs. 38.2 ± 7.4, *P* = 0.09).

Among cecal samples, the opposite pattern was observed, where age and HFD were associated with reductions in alpha diversity (young chow 465 ± 29 vs. old chow 353 ± 46, *P* = 0.001; young chow vs. young HFD 195 ± 13, *P* = 2E-9; old HFD 228 ± 43 vs. old chow 353 ± 36, *P* = 0.004). No differences in cecal alpha diversity were seen following DMM (young chow vs. young chow + DMM, 510 ± 83, *P* = 0.24). Beta diversity was significantly different among groups in both cartilage and cecal samples (*P* = 0.001 in both, Fig. 2B). The five mouse dietary and OA groups were highly segregated in cecal samples in both Beta diversity (Fig. 2B) and unsupervised clustering (Fig. 1C), with less clearly defined segregation noted among cartilage samples.

### Aging, obesity, and OA induce cartilage microbial DNA pattern alterations

**Fig. 3:**
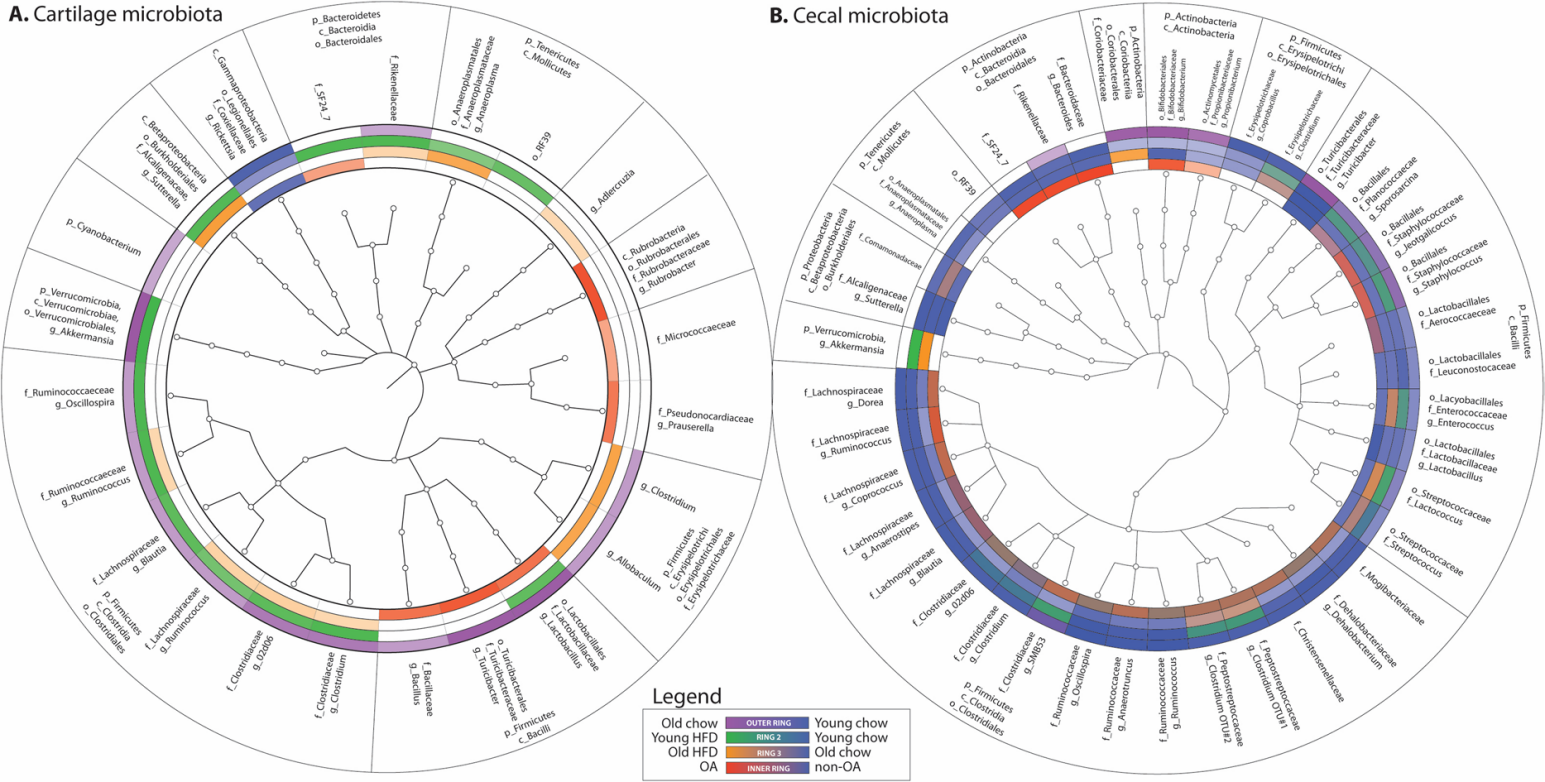
16S microbial DNA sequencing result cladograms. Only clades with statistically significant differences in LEfSe analysis under at least one age, dietary, or OA condition are represented. Color intensity represents degree of statistical significance. Outermost ring represents aging (young vs. old); ring 2 represents HFD (young HFD vs. young chow); ring 3 represents old HFD (old HFD vs. old chow); innermost ring represents OA effects (young chow + DMM vs. young chow-DMM). **A** Cartilage microbial DNA profiles. **B** Cecal microbial DNA profiles

Within cartilage, aging induced 18 clade differences (15 increased and 3 decreased in old animals vs. young) (Table 2, Supplementary Table 4, Fig. 3). HFD was associated with 34 clade differences (33 increased and 1 decreased with HFD) (Supplementary Table 5, Fig. 3). OA following DMM surgery induced 17 clade differences (15 increased in OA and 2 decreased) (Supplementary Table 6, Fig. 3). Finally, HFD in old animals was associated with 19 clade differences, all increased with HFD (Supplementary Table 7). Several cartilage clades were shared among the various conditions (Fig. 3); for example, phylum *Bacteroidetes* increased in both HFD and OA. Phylum *Firmicutes* was associated with both aging and OA, as were members of order *Turicibacterales*. Members of phylum *Verrucomicrobia* were increased in HFD and aging. Family *Coxiellaceae* within class *Gammaproteobacteria* was inversely associated with aging (enriched among young animals) and was inversely associated with OA (enriched in control animals). Certain clades including family *Rikenellaceae*, genus *Ruminococcus*, and family *Alcaligenaceae* were associated with high-fat diet in both young and old animals, whereas some clades were enriched by HFD only in old animals (members of class *Erysipelotrichales*) or by HFD only in young animals (members of order *Clostridiales*). Order *Lactobacillales* within phylum *Bacteroidetes* was enriched in aging, HFD, and OA, whereas several members of order *Clostridiales* were enriched in aging and HFD in both young and old animals.

**Table 2:** Clades altered in cecum and cartilage among age, diet, and OA groups. Values presented are linear discriminant analysis-effect size (LDA-ES). Positive values indicate increase of clade in given condition (advanced age, high-fat diet [HFD], osteoarthritis [OA]), whereas negative values indicate decrease of clade in given condition

Bacterial clade	Cecal age	Cecal HFD	Cecal OA	Cecal age + HFD	Cartilage age	Cartilage HFD	Cartilage OA	Cartilage age + HFD
*k__Bacteria.p__Actinobacteria*	4.72	−3.44						
*k__Bacteria.p__Actinobacteria.c__Actinobacteria*	4.31		3.74	−4.05				
*k__Bacteria.p__Actinobacteria.c__Actinobacteria.o__Actinomycetales.f__Micrococcaceae*							4.47	
*k__Bacteria.p__Actinobacteria.c__Actinobacteria.o__Actinomycetales.f__Propionibacteriaceae*	1.50							
*k__Bacteria.p__Actinobacteria.c__Actinobacteria.o__Actinomycetales.f__Propionibacteriaceae.g__Propionibacterium*	1.50							
*k__Bacteria.p__Actinobacteria.c__Actinobacteria.o__Actinomycetales.f__Pseudonocardiaceae*							4.56	
*k__Bacteria.p__Actinobacteria.c__Actinobacteria.o__Actinomycetales.f__Pseudonocardiaceae.g__Prauserella*							4.56	
*k__Bacteria.p__Actinobacteria.c__Actinobacteria.o__Bifidobacteriales*	4.31		3.74	−4.10				
*k__Bacteria.p__Actinobacteria.c__Actinobacteria.o__Bifidobacteriales.f__Bifidobacteriaceae*	4.31		3.74	−4.04				
*k__Bacteria.p__Actinobacteria.c__Actinobacteria.o__Bifidobacteriales.f__Bifidobacteriaceae.g__Bifidobacterium*	4.31		3.74	−4.02				
*k__Bacteria.p__Actinobacteria.c__Coriobacteriia*	4.50			4.28				
*k__Bacteria.p__Actinobacteria.c__Coriobacteriia.o__Coriobacteriales*	4.50			4.28				
*k__Bacteria.p__Actinobacteria.c__Coriobacteriia.o__Coriobacteriales.f__Coriobacteriaceae*	4.50			4.27				
*k__Bacteria.p__Actinobacteria.c__Coriobacteriia.o__Coriobacteriales.f__Coriobacteriaceae.g__Adlercreutzia*								3.06
*k__Bacteria.p__Actinobacteria.c__Rubrobacteria*							4.31	
*k__Bacteria.p__Actinobacteria.c__Rubrobacteria.o__Rubrobacterales*							4.31	
*k__Bacteria.p__Actinobacteria.c__Rubrobacteria.o__Rubrobacterales.f__Rubrobacteraceae*							4.31	
*k__Bacteria.p__Actinobacteria.c__Rubrobacteria.o__Rubrobacterales.f__Rubrobacteraceae.g__Rubrobacter*							4.31	
*k__Bacteria.p__Bacteroidetes*		−4.28	4.82	−4.55		4.63		
*k__Bacteria.p__Bacteroidetes.c__Bacteroidia*		−4.28	4.82	−4.55		4.77		
*k__Bacteria.p__Bacteroidetes.c__Bacteroidia.o__Bacteroidales*		−4.28	4.82	−4.53		4.77		
*k__Bacteria.p__Bacteroidetes.c__Bacteroidia.o__Bacteroidales.f__Bacteroidaceae*		−2.92	3.58					
*k__Bacteria.p__Bacteroidetes.c__Bacteroidia.o__Bacteroidales.f__Bacteroidaceae.g__Bacteroides*		−2.92	3.58					
*k__Bacteria.p__Bacteroidetes.c__Bacteroidia.o__Bacteroidales.f__Rikenellaceae*	4.23		4.32		4.47	4.45		3.36
*k__Bacteria.p__Bacteroidetes.c__Bacteroidia.o__Bacteroidales.f__S24_7*		−4.26	4.61			4.44	4.71	
*k__Bacteria.p__Cyanobacteria*					4.41			
*k__Bacteria.p__Firmicutes*	−5.90	−5.19		−5.04	5.23			
*k__Bacteria.p__Firmicutes.c__Bacilli*		−4.90	−5.17	−4.99				
*k__Bacteria.p__Firmicutes.c__Bacilli.o__Bacillales*	2.42	2.70	2.88					
*k__Bacteria.p__Firmicutes.c__Bacilli.o__Bacillales.f__Bacillaceae*							4.84	
*k__Bacteria.p__Firmicutes.c__Bacilli.o__Bacillales.f__Bacillaceae.g__Bacillus*							4.91	
*k__Bacteria.p__Firmicutes.c__Bacilli.o__Bacillales.f__Planococcaceae*	1.11	3.71		3.97				
*k__Bacteria.p__Firmicutes.c__Bacilli.o__Bacillales.f__Staphylococcaceae*	2.40	2.71	2.88					
*k__Bacteria.p__Firmicutes.c__Bacilli.o__Bacillales.f__Staphylococcaceae.g__Jeotgalicoccus*			2.84					
*k__Bacteria.p__Firmicutes.c__Bacilli.o__Bacillales.f__Staphylococcaceae.g__Staphylococcus*	2.40	2.71	3.36					
*k__Bacteria.p__Firmicutes.c__Bacilli.o__Lactobacillales*			−5.02		4.86	4.75		
*k__Bacteria.p__Firmicutes.c__Bacilli.o__Lactobacillales.f__Aerococcaceae*			3.57					
*k__Bacteria.p__Firmicutes.c__Bacilli.o__Lactobacillales.f__Enterococcaceae*		3.18		3.83				
*k__Bacteria.p__Firmicutes.c__Bacilli.o__Lactobacillales.f__Enterococcaceae.g__Enterococcus*		3.18		3.83				
*k__Bacteria.p__Firmicutes.c__Bacilli.o__Lactobacillales.f__Lactobacillaceae*			−5.02		4.83	4.54	4.91	
*k__Bacteria.p__Firmicutes.c__Bacilli.o__Lactobacillales.f__Lactobacillaceae.g__Lactobacillus*			−5.02		4.83	4.53	4.91	
*k__Bacteria.p__Firmicutes.c__Bacilli.o__Lactobacillales.f__Leuconostocaceae*		−4.07						
*k__Bacteria.p__Firmicutes.c__Bacilli.o__Lactobacillales.f__Streptococcaceae*		4.72		4.82				
*k__Bacteria.p__Firmicutes.c__Bacilli.o__Lactobacillales.f__Streptococcaceae.g__Lactococcus*	1.44	4.72		4.82				
*k__Bacteria.p__Firmicutes.c__Bacilli.o__Lactobacillales.f__Streptococcaceae.g__Streptococcus*		−4.00						
*k__Bacteria.p__Firmicutes.c__Bacilli.o__Turicibacterales*	5.47	−5.00	−4.63	−5.17	4.76		4.77	
*k__Bacteria.p__Firmicutes.c__Bacilli.o__Turicibacterales.f__Turicibacteraceae*	5.47	−5.00	−4.63	−5.17	4.76		4.78	
*k__Bacteria.p__Firmicutes.c__Bacilli.o__Turicibacterales.f__Turicibacteraceae.g__Turicibacter*	5.47	−5.00	−4.63	−5.17	4.76		4.77	
*k__Bacteria.p__Firmicutes.c__Clostridia*	−5.56	−4.89	4.88			5.00		
*k__Bacteria.p__Firmicutes.c__Clostridia.o__Clostridiales*	−5.56	−4.89	4.88	−4.51		5.00		
*k__Bacteria.p__Firmicutes.c__Clostridia.o__Clostridiales.f___Mogibacteriaceae*		−3.00	3.13					
*k__Bacteria.p__Firmicutes.c__Clostridia.o__Clostridiales.f__Christensenellaceae*			3.12					
*k__Bacteria.p__Firmicutes.c__Clostridia.o__Clostridiales.f__Clostridiaceae*		4.55	−4.40		4.52	4.39		
*k__Bacteria.p__Firmicutes.c__Clostridia.o__Clostridiales.f__Clostridiaceae.g__02d06*		−4.15						3.90
*k__Bacteria.p__Firmicutes.c__Clostridia.o__Clostridiales.f__Clostridiaceae.g__Clostridium*		−3.17	−3.13	−3.39		3.97		3.17
*k__Bacteria.p__Firmicutes.c__Clostridia.o__Clostridiales.f__Clostridiaceae.g__SMB53*	3.86	4.79	4.22					
*k__Bacteria.p__Firmicutes.c__Clostridia.o__Clostridiales.f__Dehalobacteriaceae*		−3.45						
*k__Bacteria.p__Firmicutes.c__Clostridia.o__Clostridiales.f__Dehalobacteriaceae.g__Dehalobacterium*		−3.45						
*k__Bacteria.p__Firmicutes.c__Clostridia.o__Clostridiales.f__Lachnospiraceae*			4.89		4.67			
*k__Bacteria.p__Firmicutes.c__Clostridia.o__Clostridiales.f__Lachnospiraceae.g___Ruminococcus*		−3.45	4.72			4.25		
*k__Bacteria.p__Firmicutes.c__Clostridia.o__Clostridiales.f__Lachnospiraceae.g__Anaerostipes*		−3.25	−4.19					
*k__Bacteria.p__Firmicutes.c__Clostridia.o__Clostridiales.f__Lachnospiraceae.g__Blautia*	−2.24	−3.25	−3.90					3.61
*k__Bacteria.p__Firmicutes.c__Clostridia.o__Clostridiales.f__Lachnospiraceae.g__Coprococcus*		−3.30		−3.31				
*k__Bacteria.p__Firmicutes.c__Clostridia.o__Clostridiales.f__Lachnospiraceae.g__Dorea*	−3.38	−3.12		−3.61				
*k__Bacteria.p__Firmicutes.c__Clostridia.o__Clostridiales.f__Peptostreptococcaceae*	3.13	3.25	2.90	3.94				
*k__Bacteria.p__Firmicutes.c__Clostridia.o__Clostridiales.f__Peptostreptococcaceae.g__Clostridium*		4.67		4.74				
*k__Bacteria.p__Firmicutes.c__Clostridia.o__Clostridiales.f__Ruminococcaceae*	−4.94	−4.57	3.32	−3.93		4.63		
*k__Bacteria.p__Firmicutes.c__Clostridia.o__Clostridiales.f__Ruminococcaceae.g__Anaerotruncus*			3.88					
*k__Bacteria.p__Firmicutes.c__Clostridia.o__Clostridiales.f__Ruminococcaceae.g__Oscillospira*	−4.86	−4.49		−3.87		4.55		
*k__Bacteria.p__Firmicutes.c__Clostridia.o__Clostridiales.f__Ruminococcaceae.g__Ruminococcus*	−3.82	−3.49				4.34		3.21
*k__Bacteria.p__Firmicutes.c__Erysipelotrichi*	−3.65							3.67
*k__Bacteria.p__Firmicutes.c__Erysipelotrichi.o__Erysipelotrichales*	−3.65							3.06
*k__Bacteria.p__Firmicutes.c__Erysipelotrichi.o__Erysipelotrichales.f__Erysipelotrichaceae*	−3.65	3.25						3.97
*k__Bacteria.p__Firmicutes.c__Erysipelotrichi.o__Erysipelotrichales.f__Erysipelotrichaceae.g__Allobaculum*								2.98
*k__Bacteria.p__Firmicutes.c__Erysipelotrichi.o__Erysipelotrichales.f__Erysipelotrichaceae.g__Clostridium*		4.53		3.84				3.89
*k__Bacteria.p__Firmicutes.c__Erysipelotrichi.o__Erysipelotrichales.f__Erysipelotrichaceae.g__Coprobacillus*	−3.29							
*k__Bacteria.p__Proteobacteria*		−4.18	−4.08	−3.97				
*k__Bacteria.p__Proteobacteria.c__Betaproteobacteria*		−4.18	−4.08	−3.92		4.37		3.53
*k__Bacteria.p__Proteobacteria.c__Betaproteobacteria.o__Burkholderiales*		−4.18	−4.08	−3.96		4.38		3.53
*k__Bacteria.p__Proteobacteria.c__Betaproteobacteria.o__Burkholderiales.f__Alcaligenaceae*		−4.18	−4.08	−3.93		4.20		4.55
*k__Bacteria.p__Proteobacteria.c__Betaproteobacteria.o__Burkholderiales.f__Alcaligenaceae.g__Sutterella*		−4.18	−4.08	−3.96		4.20		4.55
*k__Bacteria.p__Proteobacteria.c__Betaproteobacteria.o__Burkholderiales.f__Comamonadaceae*				4.30				
*k__Bacteria.p__Proteobacteria.c__Gammaproteobacteria*						−5.41		
*k__Bacteria.p__Proteobacteria.c__Gammaproteobacteria.o__Legionellales*					−5.35			
*k__Bacteria.p__Proteobacteria.c__Gammaproteobacteria.o__Legionellales.f__Coxiellaceae*					−5.35		−5.38	
*k__Bacteria.p__Proteobacteria.c__Gammaproteobacteria.o__Legionellales.f__Coxiellaceae.g__Rickettsiella*					−5.35		−5.38	
*k__Bacteria.p__Tenericutes*		−3.52		−3.35		4.23		
*k__Bacteria.p__Tenericutes.c__Mollicutes*		−3.52		−3.35		4.23		
*k__Bacteria.p__Tenericutes.c__Mollicutes.o__Anaeroplasmatales*		−2.86						3.89
*k__Bacteria.p__Tenericutes.c__Mollicutes.o__Anaeroplasmatales.f__Anaeroplasmataceae*		−2.86						3.89
*k__Bacteria.p__Tenericutes.c__Mollicutes.o__Anaeroplasmatales.f__Anaeroplasmataceae.g__Anaeroplasma*		−2.86						3.88
*k__Bacteria.p__Tenericutes.c__Mollicutes.o__RF39*		−3.50		−3.34		4.15		
*k__Bacteria.p__Verrucomicrobia*		5.29		5.14	4.73	4.77		
*k__Bacteria.p__Verrucomicrobia.c__Verrucomicrobiae*		5.29		5.14	4.72	4.77		
*k__Bacteria.p__Verrucomicrobia.c__Verrucomicrobiae.o__Verrucomicrobiales*		5.29		5.16	4.72	4.77		
*k__Bacteria.p__Verrucomicrobia.c__Verrucomicrobiae.o__Verrucomicrobiales.f__Verrucomicrobiaceae*		5.29		5.14	4.72	4.77		
*k__Bacteria.p__Verrucomicrobia.c__Verrucomicrobiae.o__Verrucomicrobiales.f__Verrucomicrobiaceae.g__Akkermansia*		5.29		5.16	4.72	4.77

*Cecal microbiome differences are associated with aging, obesity, and OA*

Within cecal samples, aging induced 36 microbiome clade changes, with 23 clades increased with aging and 13 decreased with aging. HFD induced 59 changes, 19 clades increased and 40 decreased with HFD (Table 2, Supplementary Tables 8-11). OA was associated with 41 clade changes; 26 clades increased and 15 clades diminished. In aged animals, HFD induced 43 clade differences; 17 enriched in HFD and 26 enriched in chow. Similar to our cartilage findings, a number of clades were shared among groups (Fig. 3). Class *Actinobacteria* was increased in aging and OA, particularly order *Bifidobacteriales* within this class. Family *Rikenellaceae* was associated with aging and OA, whereas order *Bacillales* were increased in aging, HFD, and OA. Similarly, members of order *Clostridiales*, including family *Peptostreptococcaceae* and genus *SMB53* were increased in aging, HFD, and OA. Genus *Staphylococcus* was increased in aging, HFD, and OA, whereas genus *Lactococcus* was increased in both aging and HFD. Also similar to our cartilage findings, certain clades were associated with HFD only in aged animals, including members of class *Coriobacteriia*. Genus *Blautia* within family *Lachnospiraceae* were decreased in all 3 conditions: aging, HFD, and OA. We found opposing changes among family *Lactobacillus*, however, where increases were associated with aging, HFD, and OA in cartilage samples, whereas decreases were associated with aging, HFD, OA, and aged HFD in cecal samples.

### HFD induces a shift in the cecal microbiome towards increased Gram-negative constituents

Next, we queried the proportion of constituent microbial DNA from Gram-negative organisms among the various age, diet, and OA groups, as we had previously identified increases in Gram-negative fractions in our human OA cartilage 16S work [8]. In the present study, no differences were seen between groups in cartilage samples. However, in cecal data, there were increases in Gram-negative fraction in both young and old HFD (young HFD 50 ± 2% vs. young chow 18 ± 4%, mean ± SEM, *P* = 4.6E-5; old HFD 50 ± 4% vs. 28 ± 2%, *P* = 0.003) and a nonsignificant increased Gram-negative fraction in aging (old chow 28 ± 2% vs. young chow: 18 ± 4%, *P* = 0.08). No differences were seen in Gram-negative fraction in post-DMM OA cecal samples compared to non-OA controls (*P* = 0.2).

### Clade-specific qPCR confirmed alterations of microbiota in cartilage and cecum with aging, obesity, and OA in an independent mouse cohort

**Fig. 4:**
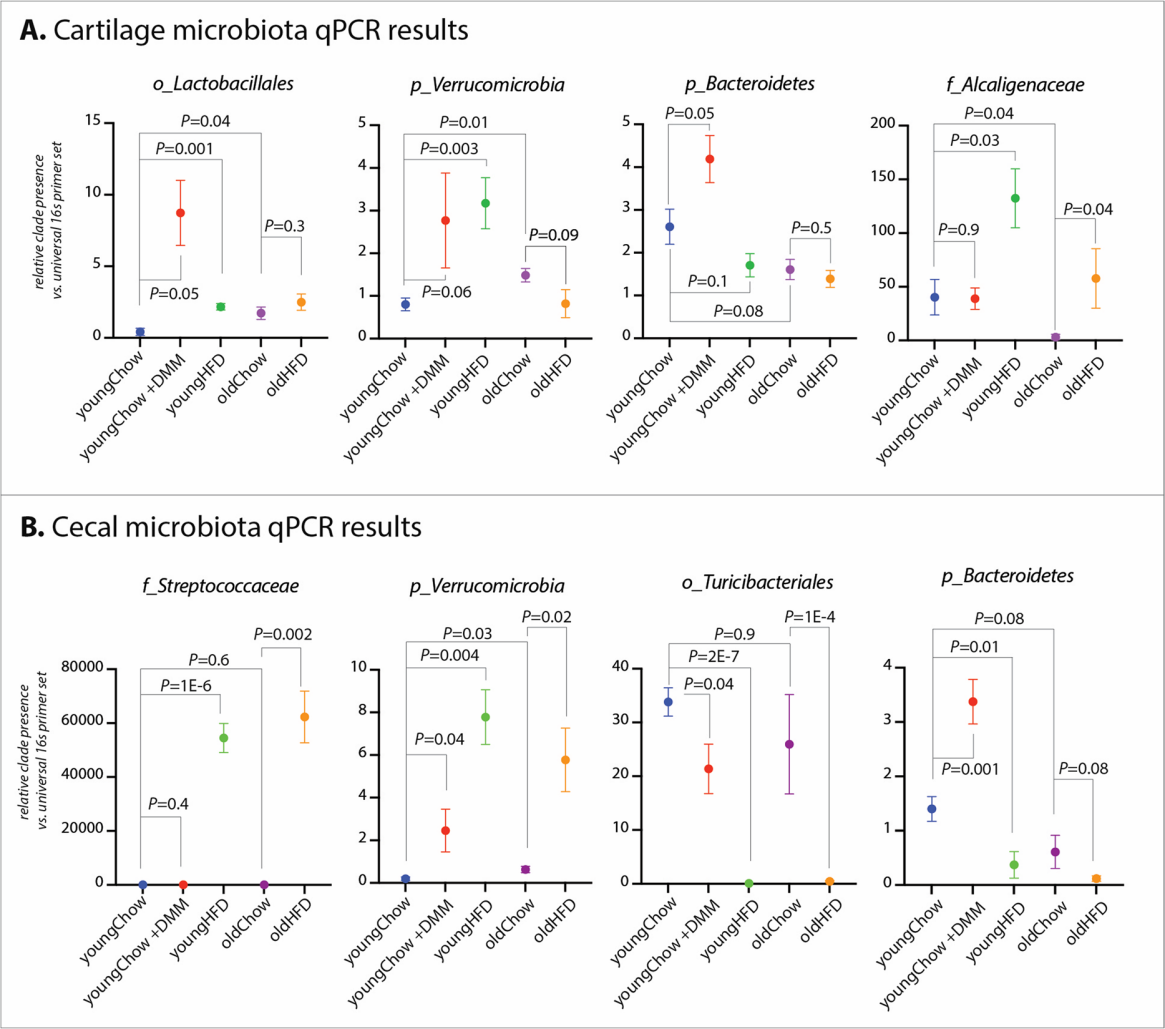
Confirmation of microbiome 16S deep sequencing results in an independent animal cohort using clade-specific qPCR. Vertical axis represents relative clade presence vs. universal 16S primer set. **A** Cartilage qPCR results. **B** Cecal qPCR results

We next confirmed our findings in a separate cohort of animals from each condition (*n* = 6 young chow, *n* = 6 young HFD, *n* = 6 old chow, *n* = 6 young chow + DMM) (Fig. 4) using previously published clade-specific qPCR protocols. These qPCR results confirmed our deep-sequencing analysis. Specifically, within cartilage, we confirmed increases of order *Lactobacillus* in aging (*P* = 0.04), HFD (*P* = 0.001), and OA (*P =* 0.05). Phylum *Verrucomicrobia* was increased in HFD (*P* = 0.003) and aging (*P* = 0.01), with a nonsignificant increase in OA (*P* = 0.06). Phylum *Bacteroidetes* was increased in OA (*P* = 0.05). Family *Alcaligenaceae* was increased in HFD in both young (*P* = 0.03) and aged (*P* = 0.04) animals.

Within cecal samples, family *Streptococcaceae* was increased with HFD in both young (*P* = 1E-6) and old (*P* = 0.002) mice. Phylum *Verrucomicrobia* was increased in HFD (*P* = 0.004), aging (*P* = 0.03), HFD in old animals (*P =* 0.02), and OA (*P* = 0.04, *Verrucomicrobia* did not reach statistical significance in cecal 16S-OA data). Order *Turicibacteriales* was reduced in OA (*P* = 0.04), reduced in HFD (*P* = 2E-7), and reduced in HFD treatment in aged animals (*P* = 1E-4). Finally, phylum *Bacteroidetes* was increased in OA (*P* = 0.001) but decreased with HFD in young (*P* = 0.01) and old (*P* = 0.08) animals, with a nonsignificant decrease in aging (*P* = 0.08).

### Metagenomes imputed from 16s data suggest alterations in several canonical bacterial pathways with diet, aging, and OA in cartilage and cecum

**Fig. 5:**
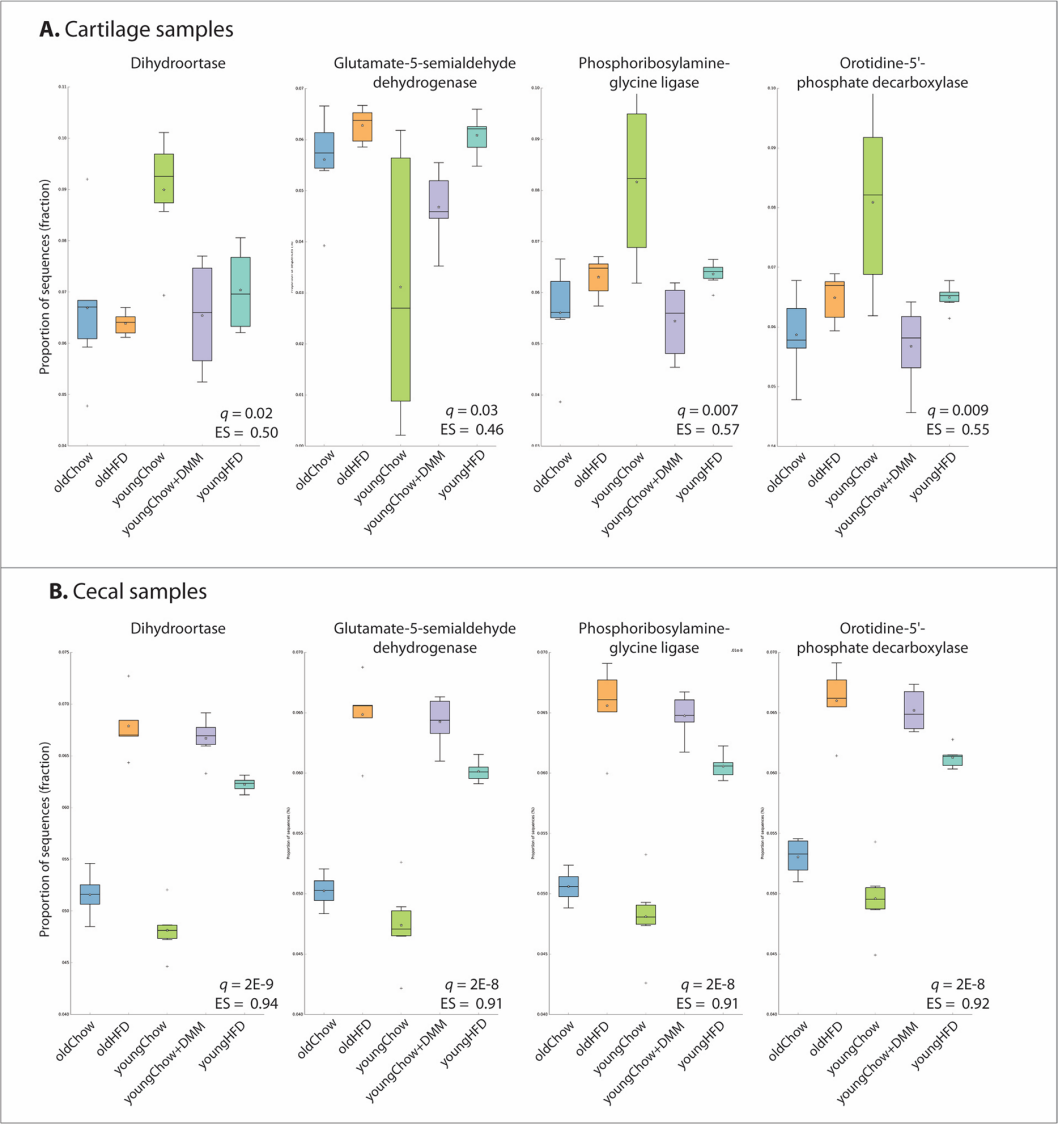
Functional metagenome canonical pathways imputed using PICRUSt from 16S sequencing data. Effect size (ES) calculated using Eta-squared. Statistical significance calculated using ANOVA with Benjamini-Hochberg multiple test correction (*q* values). Bars represent mean ± SD, stars represent mean. Most significant pathways shared among both cartilage and cecal samples are presented. **A** Cartilage sample data. **B** Cecal sample data

Given the significant clade differences we found above in cartilage and cecal microbiota, we estimated whether differences in bacterial metagenomes might also exist among the mouse groups. To do this, we imputed metagenome function using the Phylogenetic Investigation of Communities by Reconstruction of Unobserved States (PICRUSt) package [23]. Indeed, within cartilage, we identified enrichment in 897 Kyoto Encyclopedia of Gene and Genomes (KEGG) pathways (Table 3, Supplementary Table 12) and 2631 KEGG pathways significantly different among groups in cecal samples (Table 3, Supplementary Table 13). Approximately 37% of these pathways were shared in cecal and cartilage groups (339 of 896) (Table 3, Fig. 5, Supplementary Table 14), including dihydroorotase (*q* = 0.02 in cartilage, *q* = 2E-9 in cecal, decreased in aging, HFD, and OA vs. control) and glutamate-5-semialdehyde dehydrogenase (*q* = 0.03 in cartilage, *q* = 2E-8 in cecal), decreased in control and increased in other groups.

**Table 3:** Top imputed metagenomes of cartilage and cecal clade variations among aging, diet, and OA groups

KEGG pathway	*q* value (BH corrected)		Effect size	
Cartilage microbiota (top 15 of 896)				
Membrane dipeptidase	8E-05		0.82	
Methylthioribose-1-phosphate isomerase	9E-05		0.81	
Putative acetyltransferase	0.0002		0.78	
Histidinol-phosphatase (PHP family)	0.0002		0.77	
DNA (cytosine-5-)-methyltransferase	0.0003		0.77	
Carnitine O-acetyltransferase	0.0003		0.77	
DNA adenine methylase	0.0004		0.75	
Cd2+/Zn2+-exporting ATPase	0.0004		0.75	
Carboxynorspermidine decarboxylase	0.0005		0.74	
Uroporphyrin-III C-methyltransferase	0.0005		0.74	
D-alanyl-D-alanine carboxypeptidase / D-alanyl-D-alanine-endopeptidase (penicillin-binding protein 4)	0.0005		0.74	
Putative SAM-dependent methyltransferase;ribosomal RNA large subunit methyltransferase I	0.0005		0.75	
RNA polymerase sigma-70 factor, ECF subfamily	0.0005		0.73	
Phosphopantothenoylcysteine decarboxylase / phosphopantothenate--cysteine ligase	0.0006		0.73	
Thiamine biosynthesis lipoprotein	0.0006		0.73	
Cecal microbiota (top 15 of 2631)				
Dihydroorotase	2E-09		0.94	
ATP-dependent DNA helicase DinG	6E-09		0.93	
Phosphoribosylformylglycinamidine synthase	2E-08		0.92	
Glutamate-5-semialdehyde dehydrogenase	2E-08		0.91	
Glutamate 5-kinase	2E-08		0.91	
Phosphoribosylamine--glycine ligase	2E-08		0.91	
Orotidine-5′-phosphate decarboxylase	2E-08		0.92	
Glycerol uptake facilitator protein	2E-08		0.91	
Oligo-1,6-glucosidase	2E-08		0.92	
Aspartate carbamoyltransferase catalytic subunit	2E-08		0.91	
Electron transport complex protein RnfA	2E-08		0.90	
NADH dehydrogenase	2E-08		0.90	
Electron transport complex protein RnfE	3E-08		0.90	
Cell filamentation protein	3E-08		0.91	
Thioredoxin 1	3E-08		0.90	
KEGG pathway	*q* value cartilage (BH corrected)	Effect size cartilage	*q* value cecal (BH corrected)	Effect size cecal
Imputed metagenomic pathways shared by both cartilage and cecal microbiota (top 15 of 339)				
Dihydroorotase	0.02	0.50	2E-09	0.94
Glutamate-5-semialdehyde dehydrogenase	0.03	0.46	2E-08	0.91
Glutamate 5-kinase	0.03	0.46	2E-08	0.91
Phosphoribosylamine--glycine ligase	0.007	0.57	2E-08	0.91
Orotidine-5′-phosphate decarboxylase	0.009	0.55	2E-08	0.92
Aspartate carbamoyltransferase catalytic subunit	0.01	0.53	2E-08	0.91
Electron transport complex protein RnfA	0.04	0.44	2E-08	0.90
Thioredoxin 1	0.02	0.49	3E-08	0.90
Electron transport complex protein RnfC	0.04	0.44	3E-08	0.90
Aspartyl-tRNA(Asn)/glutamyl-tRNA (Gln) amidotransferase subunit B	0.02	0.50	3E-08	0.90
Stage V sporulation protein B	0.02	0.49	3E-08	0.90
Stage V sporulation protein AC	0.01	0.52	3E-08	0.90
Electron transport complex protein RnfD	0.05	0.43	3E-08	0.90
Stage II sporulation protein D	0.004	0.59	3E-08	0.90
Stage V sporulation protein AE	0.02	0.50	3E-08	0.90

## Discussion

In this study, we first investigated the gut microbiome origins of cartilage microbial DNA patterns, then performed an analysis of alterations in these signatures associated with non-genetic OA risk factors that change the gut microbiome, including age, obesity associated with high-fat diet (HFD), and OA following DMM surgery using mouse models. These new data expand upon our initial identification of microbial DNA patterns within human cartilage and young OA-susceptible and OA-resistant mouse cartilage [8] and offer a new perspective on the development and plasticity of articular microbial DNA signatures, as well as demonstrating both cecal and cartilage microbial DNA changes associated with DMM-induced OA without other risk factors present.

First, we found cartilage microbial DNA patterns develop rapidly within 48 h following inoculation of GF mice with a gut microbiome. This development exhibited an exponential plateau pattern, which would be expected of microbes populating a size-confined location and argues against any potential contamination, which would be expected to have a similar 16s microbial sequencing read number across all timepoints. These findings bolster the hypothesis that certain clades of cartilage microbes and/or microbial DNA arise through gut permeability. Changes in gut permeability have been an area of interest in OA [32] given previous descriptions of increased bacterial translocation in obese human OA patients [33, 34]. Indeed, in the current study, we found indirect evidence for increases in gut permeability in HFD and OA, although not with aging. Future work should expand upon our findings by tracing microbes and microbial products during the initial phases (0–48 h) of this seeding to better evaluate the precise route of inoculation (e.g., via blood-synovium-synovial fluid-cartilage, or via subchondral bone). We must also consider trafficking from other mucosal sites, including the lung or oral cavity, as has been described in the context of other autoimmune rheumatic diseases [35, 36].

Next, we found several cartilage and cecal microbiome alterations associated with OA and individual OA risk factors. Specifically, we identified increases in cartilage microbial alpha diversity with aging, HFD, and OA and the opposite pattern within cecal samples, where decreases in alpha diversity were noted with aging and HFD (but not OA). We found significant differences between groups in beta diversity in both cartilage and cecal samples. Our finding of increased alpha diversity within cartilage associated with OA risk factors and OA itself is curious, particularly because the OA mouse cartilage used in this experiment represented early-stage disease, 4 weeks after DMM surgery. In our previous human cartilage microbial work, we identified decreases in microbial alpha diversity, albeit in end-stage OA tissue. This increased cartilage microbial diversity in aging, HFD, and early OA may reflect transient increases in intestinal permeability and bacterial translation into the systemic circulation and articular deposition; future studies should expand our work to include later timepoints. The decreases in alpha diversity were found in cecal samples with both aging and HFD mirror previous human findings [37, 38].

Our data add to a growing body of literature linking alterations in various microbiome niches with OA in both human patients and mouse models. Unlike previous human cohorts, however, we were able to assess microbial pattern changes associated with risk factors and OA individually. In the LifeLines-DEEP and Dutch Rotterdam (RSIII) human OA cohorts, Boer et al. identified four bacterial clades associated with knee pain in 16S analysis of the fecal microbiome, including class *Bacilli*, order *Lactobacillales*, family *Streptococcaceae*, and genus *Streptococcus* [39]. We found evidence for both increases and decreases in various members of *Lactobacillales* within cecal samples: HFD consistently resulted in increases in family *Enterococcaceae*, whereas decreases were noted in DMM animals within family *Lactobacillaceae*. Within cartilage, we found increases in family *Lactobacillaceae* in aging, HFD, and DMM conditions, agreeing with the Boer findings. Further, we found family *Streptococcceae* increased in cecum with HFD in both young and old animals. Of note, members of *Streptococcus* have been associated with gut, oral, or synovial tissues of OA patients in 5 previous studies [36, 39–42]; our data suggest that these *Streptococcus* associations may represent an effect of obesity rather than either aging or OA independently. Additionally, *Streptococcus* is a known component of the oropharyngeal flora and, thus, future work should confirm the location of inoculation for this particular species and profiles associated with the oropharyngeal microbiome.

In 2018, Schott et al. demonstrated that oligofructose prebiotic supplementation reduced histologic OA following DMM of HFD-treated mice [7]. *Actinobacteria* were reduced in non-treated animals; we saw similar decreases in *Actinobacteria* in cecal samples in HFD mice. In 2018, Zhao and colleagues published an analysis of synovial fluid and synovial tissue from knees of human OA and RA patients [42]. Many of the bacterial DNA clades they found characteristic of knee OA synovial tissue were also found in cartilage in the present study. These included increases in family *Clostridiaceae* with both aging and HFD, genus *02d06* in aged HFD animals, genus *Clostridium* with HFD in both young and old animals, family *Lachnospiraceae* in aging, genus *Ruminococcus* with HFD, and genus *Blautia* in aged HFD animals. OA-associated increases in *Clostridium* species have been previously described in the gut microbiome in 3 human [40, 43, 44] and 2 rat OA model [45, 46] studies; our data suggest these increases may be related to both aging and HFD. In 2020, Song et al. published a study of *Lactobacillus* M5 supplementation in an HFD-induced OA mouse model, noting decreases in OA pathologic scores following supplementation. In fecal 16S analysis, they noted a strong positive correlation between the presence of *Ruminococcus*, *Streptococcus*, and *Lactococcus* and OA histopathology scores [47]; in our data, all 3 of these clades were associated with HFD and/or aging in cartilage samples. Curiously, however, *Ruminococcus* was reduced in cecal samples with aging and HFD, and *Streptococcus* was reduced in cecal samples with HFD. *Lactococcus* was increased with both aging and HFD in cecal samples, mirroring our cartilage findings.

We also identified shifts both in Gram-negative proportion in HFD and *Firmicutes:Bacteroidetes* ratio in HFD and OA. Finally, we imputed functional metagenomic data from our 16S sequencing and identified several differentially expressed canonical pathways within cecal and cartilage microbiota constituents. Increases in the *Firmicutes*:*Bacteroidetes* (F:B) ratio in the fecal microbiome have been linked to obesity-related dysbiosis and aging in the human microbiome [48, 49], and an increase in ratio has been demonstrated in OA patients in several studies within the gut [50, 51] and synovial tissue [42], as well as fecal samples in both rat [46] and mouse OA models [52]. We found an increase in F:B ratio in cecal samples associated with HFD in both young and old animals but identified an intriguing decrease in F:B ratio in both cecal samples and cartilage with OA.

Previous microbiome association studies in OA have operated under the assumption that the microbiome:OA causal association is unidirectional; that is, alterations in the host microbiome drive OA susceptibility. Relatively little attention has been given to the possibility that OA itself may induce modifications of the host microbiome by as-yet unidentified mechanisms, potentially including systemic inflammation, pain-related dietary changes, etc. In the present study, we included a comparison of microbiota profiling from both OA and non-OA animals that allow us an early insight into this question. We found that the induction of post-traumatic OA via DMM surgery resulted in significant shifts in the cartilage microbial DNA patterns and also, unexpectedly, in the cecal microbiome. OA samples clustered most closely to chow-fed controls (both young and old, Fig. 2C).

Individual OA-associated clade changes in cartilage were generally concordant with other OA risk factors; for example, increases in genus *Lactobacillus* were associated with OA, HFD, and aging (Fig. 3A). However, several cecal microbiome changes were discordant in OA and OA risk factors; for example, genus *Bacteroides* was increased in OA but decreased with HFD in both young and old animals (Fig. 3B), similar results were noted within multiple genera of family *Lachnospiraceae*. We also noted some discordance in microbial signatures in cartilage compared to cecal samples; for example, members of order *Clostridiales* tended to be increased with aging and HFD in cartilage but decreased in cecal samples, members of phylum *Proteobacteria* were generally decreased with OA risk factors in cartilage but increased in cecum. These differences indicate that cartilage microbial patterns do not exclusively reflect the gut microbiome; most likely both systemic and local immune responses both in the gut and peripheral joints function to “filter” circulating microbes and/or microbial DNA. This likely would occur through a combination of mechanisms, including species-specific immunological recognition and clearing of particular species and/or global reduction or increase in immunoregulatory cells (previously shown to be directly affected by gut microbiome-produced metabolic products [53]), alteration of gut permeability to both bacteria and metabolites through loosening or tightening of tight junction proteins, etc. Further characterization of these species-specific immune responses and any changes associated with OA or OA risk factors should be the focus of future research efforts. Intriguingly, surgery itself may have some impact on the microbiome. One previous study correlated gut microbiome disturbances to surgical interventions, where abdominal surgery in mice results in gut microbiome disturbance, correlated with alterations in microbiome-derived metabolic products that then are associated with post-operative cognitive dysfunction [54].

Our bacterial functional analysis, performed by reconstructing metagenomes using PICRUSt, identified pathways associated with OA, HFD, and aging (Table 3, Fig. 4, Supplementary Table 14). Several of these pathways are consistent with previous OA reports. Dihydroorotase, catalyzing an important step in pyrimidine biosynthesis, was found in both cartilage (ES = 0.5, *q* = 0.02) and cecal (ES = 0.94, *q* = 2E-9) samples and has been previously associated with hip OA [55]. Glutamate-5-semialdehyde dehydrogenase was similarly associated in both cartilage and cecal samples; this enzyme has been identified as a potential urine biomarker of OA [56]. Rushing et al. recently characterized fecal metabolomic profiles from hand and knee OA patients and identified 6 metabolic pathways associated with OA [57]; in our analysis, we found 2 of these associated with OA and OA risk factors, including phosphoribosylamine-glycine ligase, an enzyme catalyzing the second step of purine biosynthesis, and pyruvate orthophosphate dikinase.

Our study does have several limitations. We evaluated cartilage and cecal microbiota profiles only in male mice, as only male mice exhibit a reliable OA phenotype following DMM surgery [11]. Future studies should evaluate sex differences in both cartilage and gut microbiota profiles and determine the plasticity of these profiles following microbiome modification. This pilot study also included relatively few animals; however, we included an independent cohort that confirmed many of our findings; future studies should expand upon our numbers and allow for meta-analysis confirmation of our findings.

Sensitive sequencing analysis of bacterial samples always carries the possibility of environmental contamination during processing, particularly in cartilage samples that have a relatively low amount of microbial DNA present. To reduce this risk, we implemented a rigorous decontamination protocol including processing samples in a sterile environment, decontaminating PCR reagents and plasticware both enzymatically and with UV light, and have previously demonstrated only minimal amplification of contaminating microorganisms following these procedures in germ-free animals [8]. Furthermore, all samples were processed in parallel, increasing the possibility that any differences seen were reflective of underlying biological signals rather than contamination. Finally, as is the case with all DNA sequencing-based microbiome analyses, we characterized bacterial nucleic acids and therefore do not have data regarding the presence of living organisms present in a sample.

Future OA microbiome studies should expand to include sex differences and functional analyses to identify the mechanism(s) whereby articular tissues are inoculated with microbial sequences and/or living microorganisms. Additionally, the “gatekeeper” mechanisms, likely immune in nature, that allow for the deposition of microbial sequences in cartilage from some bacterial clades but not others should be elucidated. These studies may offer insight into why only certain microbial clades exhibited similar shifts with HFD, aging, and OA in both cecal and cartilage tissues. Future OA animal studies should also be mindful of the potential for microbiome modification induced by OA itself and ensure that microbiome shifts they identify are a result of the tested intervention and not the progression of the underlying disease. Additional tissues should be evaluated in a similar way to our current study, including infrapatellar fat pad and/or synovium, to increase our understanding of the diversity of murine joint tissue microbial DNA sequences. From a pathophysiological/seeding standpoint, direct analysis of intestinal permeability and quantitation of tight junction proteins within the gut would be of importance, as would be correlations between the gut/cartilage microbiota and both systemic and joint-specific immunophenotypes and cytokine patterns. Finally, future work to modify the microbiome will be necessary to determine whether potentially pathogenic clades may be intentionally depleted in cecum and/or cartilage; these investigations may provide a novel avenue for the development of future microbiome-targeted OA therapeutics.

### Supplementary information

ESM 1 (XLSX 214 kb)

ESM 2 (PNG 96 kb)

High resolution image (TIFF 19110 kb)

ESM 3 (PNG 151 kb)

High resolution image (TIFF 9064 kb)
