# Supplementary figures and images for: Murine cartilage microbial DNA deposition occurs rapidly following the introduction of a gut microbiome and changes with obesity, aging, and knee osteoarthritis
Source: GeroScience. 2023 Nov 9;46(2):2317–41. doi: 10.1007/s11357-023-01004-z (PMC10828335; doi:10.1007/s11357-023-01004-z)

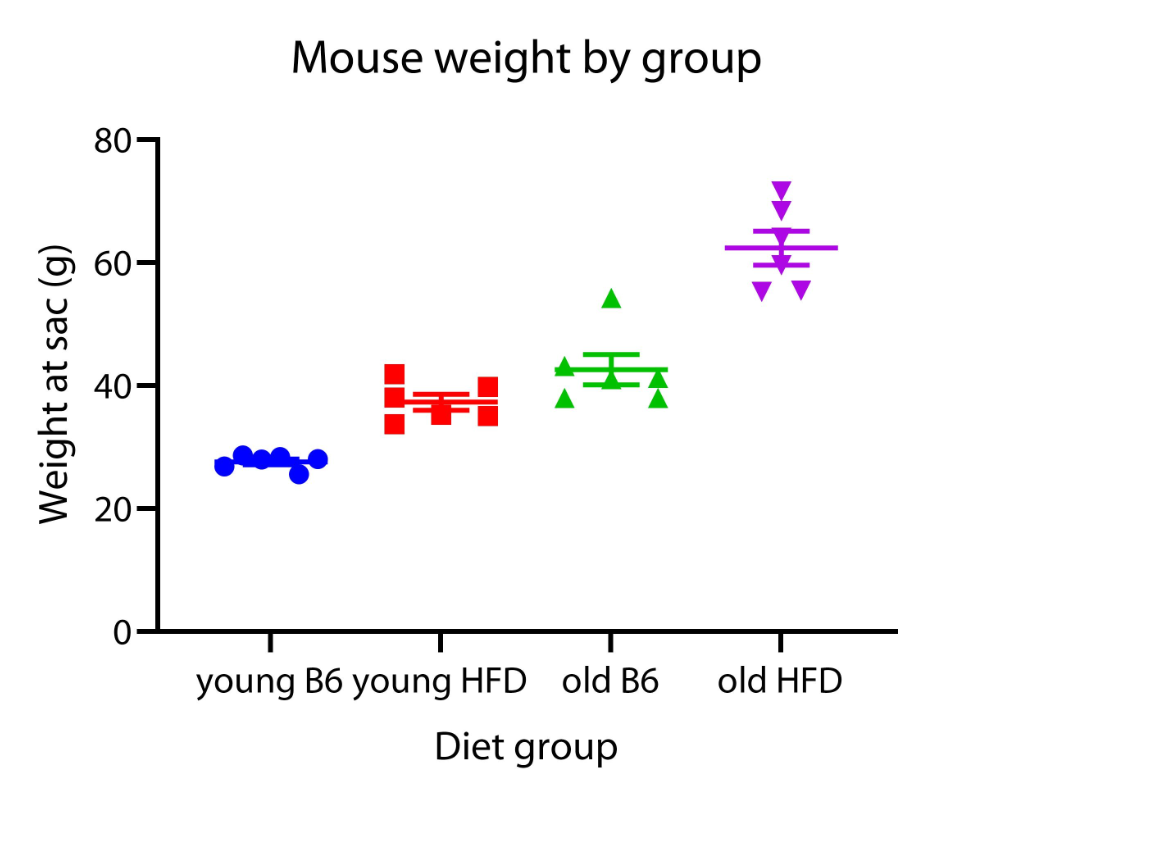

Supplement: Supplementary file 2 — (PNG 96 kb) [file 11357_2023_1004_Fig6_ESM.png]

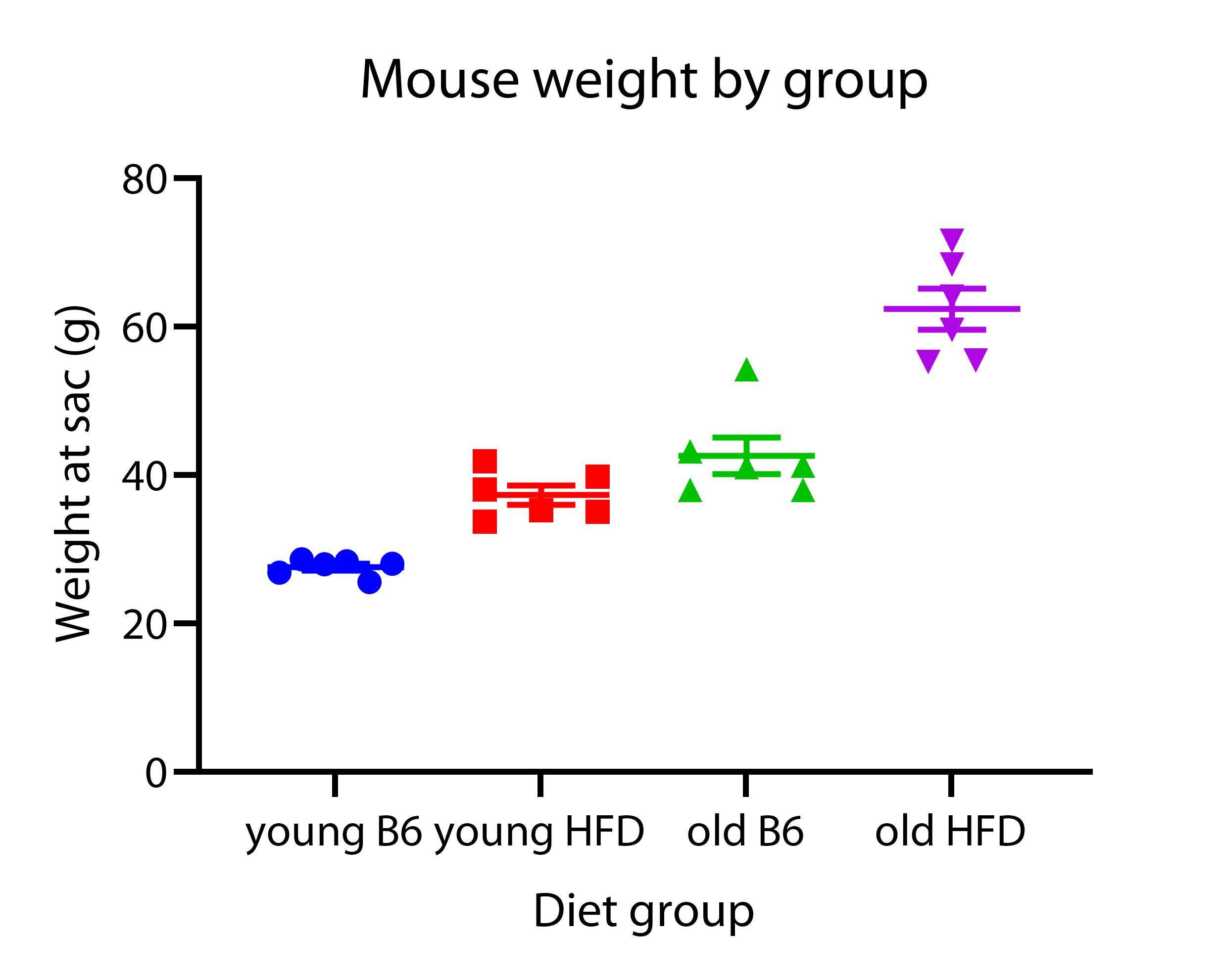

Supplement: Supplementary file 3 — High resolution image (TIFF 19110 kb) [file 11357_2023_1004_MOESM2_ESM.tiff]

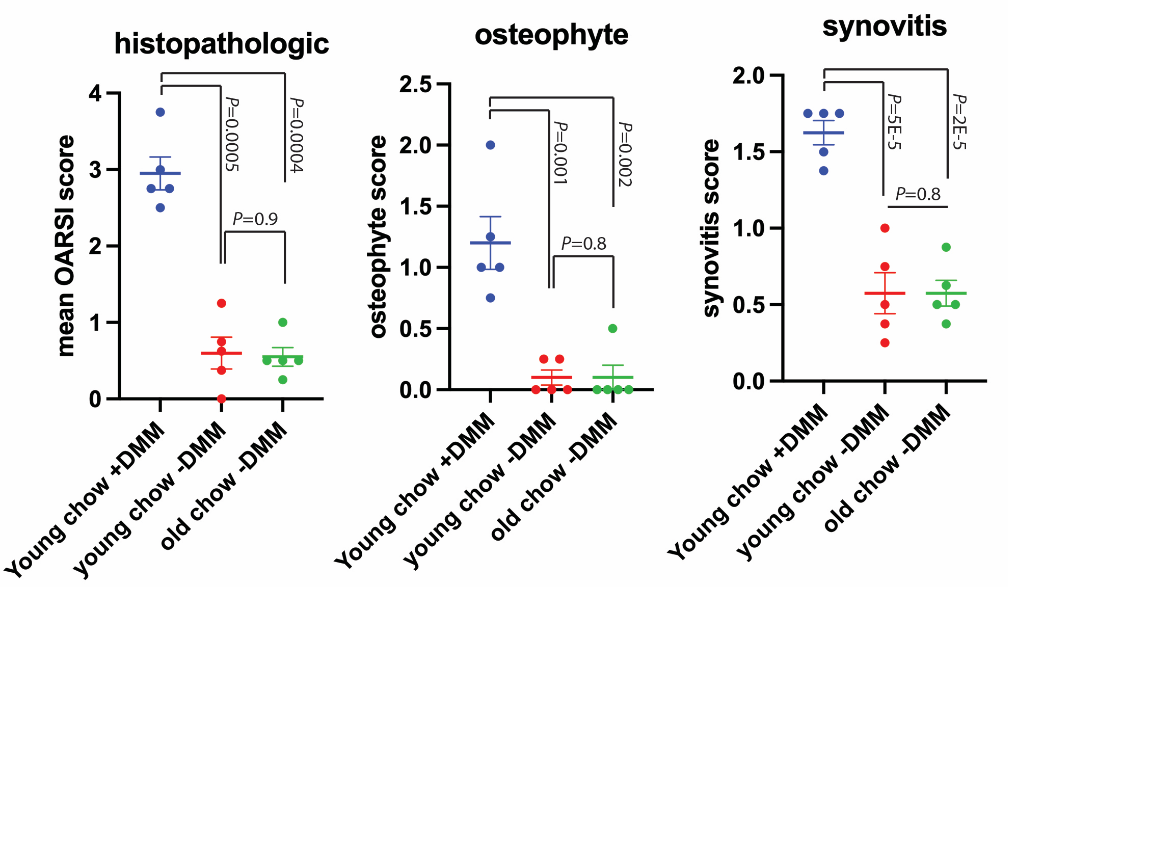

Supplement: Supplementary file 4 — (PNG 151 kb) [file 11357_2023_1004_Fig7_ESM.png]

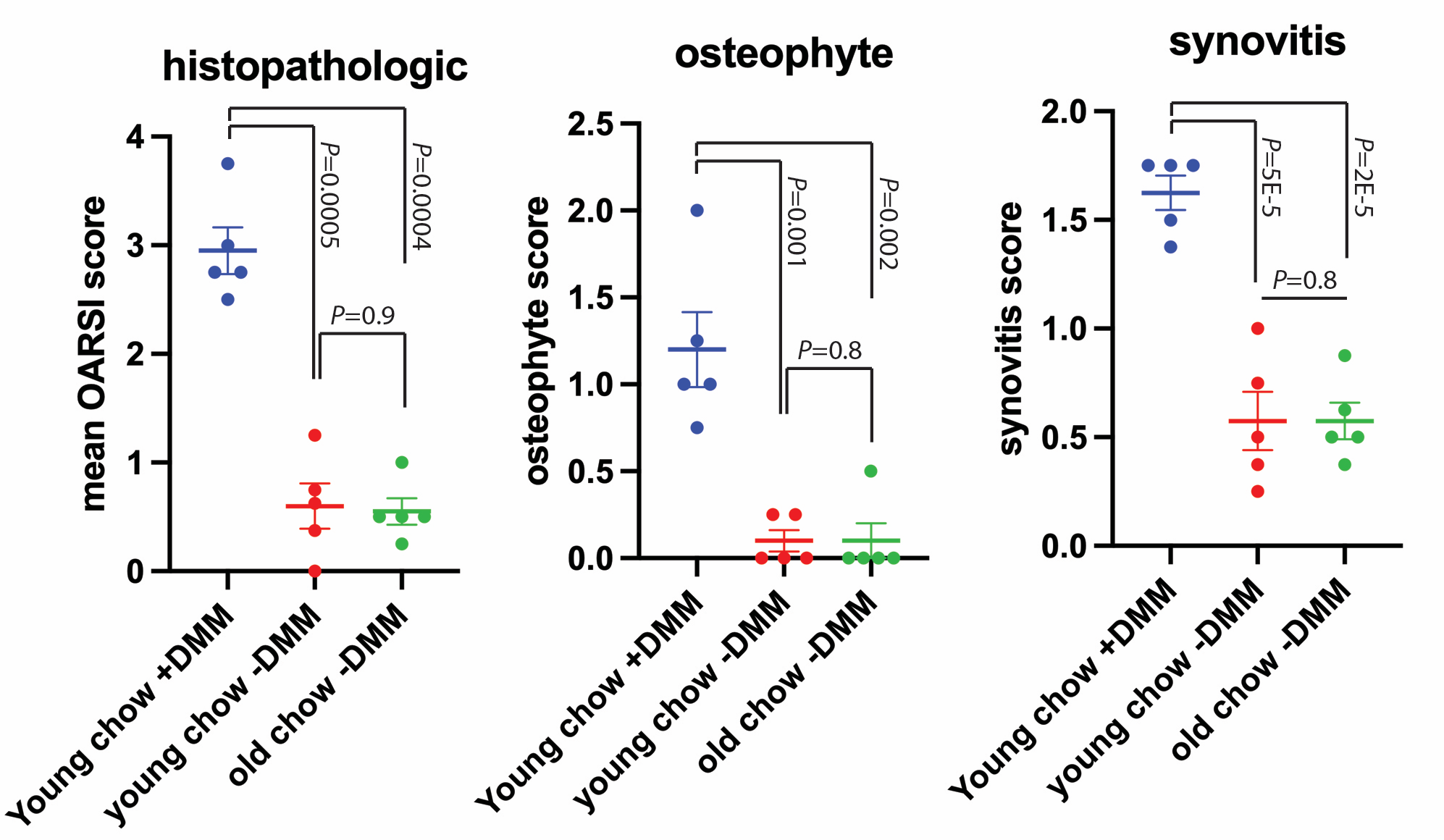

Supplement: Supplementary file 5 — High resolution image (TIFF 9064 kb) [file 11357_2023_1004_MOESM3_ESM.tiff]
